# Comparing the Performance of McMaster, FLOTAC and Mini-FLOTAC Techniques in the Diagnosis of Strongylid Infections in Two Horse Populations in Portugal

**DOI:** 10.3390/pathogens14111075

**Published:** 2025-10-22

**Authors:** Marta Varandas, João Lozano, Ricardo Agrícola, Lídia Gomes, Teresa Rosa, Mariana Magalhães, Luís Lamas, Laura Rinaldi, Manuela Oliveira, Adolfo Paz-Silva, Luís Madeira de Carvalho

**Affiliations:** 1 Centre for Interdisciplinary Research in Animal Health (CIISA), Faculty of Veterinary Medicine, University of Lisbon, 1300-477 Lisbon, Portugalmoliveira@fmv.ulisboa.pt (M.O.); 2 Associate Laboratory for Animal and Veterinary Sciences (AL4AnimalS), 1300-477 Lisbon, Portugal; 3 Veterinary Hospital, Faculty of Veterinary Medicine, Lusófona University, 1749-024 Lisbon, Portugal; 4 Department of Veterinary Medicine and Animal Production, University of Naples Federico II, 80137 Naples, Italy; 5 Centre for Ecology, Evolution and Environmental Changes (cE3c), Global Change and Sustainability Institute (CHANGE), Faculty of Sciences, University of Lisbon, 1749-016 Lisbon, Portugal; 6 Control of Parasites Research Group (COPAR, GI-2120), Department of Animal Pathology, Faculty of Veterinary, University of Santiago de Compostela, 27002 Lugo, Spain

**Keywords:** equine, gastrointestinal parasites, coprological diagnosis, FLOTAC, Mini-FLOTAC, McMaster

## Abstract

The diagnosis of gastrointestinal (GI) strongyle infections in equids is still mainly performed using quantitative coprological techniques, like the McMaster (McM), but more sensitive and precise techniques, like FLOTAC (FL) and Mini-FLOTAC (MF), have been proposed over the past 20 years. The present study aimed to compare the analytical performance of these three methods in the diagnosis of strongyle infections in horses. Between October 2023 and June 2024, 32 fecal samples were processed using the McM, FL and MF techniques to identify strongyles’ eggs, estimate their shedding (eggs per gram of feces, EPG), standard errors, sensitivities, precisions, and perform Spearman’s correlation and Cohen’s kappa analyses. The McM detected a higher shedding (584 ± 179 EPG), in comparison with FL and MF, with both these differences being statistically significant (*p* < 0.001); FL achieved the highest precision (72%), which differed significantly from McM (*p* = 0.03). All techniques were positively (r_s_ = 0.92–0.96) and significantly (*p* < 0.001) correlated and shared substantial (k = 0.67–0.76) and significant (*p* < 0.001) agreement. The MF achieved the highest diagnostic sensitivity (93%), followed by FL (89%) and McM (85%), although not significantly (*p* = 0.90). These results suggest the usefulness of implementing FL or MF methods in equine medicine for precise and, in the latter case, quick parasitological diagnosis.

## 1. Introduction

Strongylid nematodes are ubiquitous gastrointestinal (GI) parasites of most horse populations worldwide and have the potential to represent a serious challenge to equine health and welfare, particularly large strongyles like *Strongylus* spp. and *Triodontophorus* spp. and small strongyles such as *Cyathostomum* spp., *Cylicocyclus* spp. and *Cylicostephanus* spp. [1,2,3,4]. Some clinical signs include colic, diarrhea, loss of appetite and emaciation, reduced performance, impaired growth, anemia, and rough hair coat [5,6,7,8,9].

To control them, many farms still rely on periodical administrations of antiparasitic drugs without a previous laboratorial diagnosis, which is an unsustainable approach on a long-term basis, often leading to the development of antiparasitic drug resistance [7,9,10,11,12,13,14,15,16,17,18]. Successful and sustainable antiparasitic control programs can be achieved by reliable diagnostic tools, like fecal egg count (FEC) methods, which allow the detection of GI parasitic infections, an estimation of their intensity, and an assessment of antiparasitic drug treatment effectiveness [7,9,19,20,21,22,23,24,25,26]. There are several FEC methods that differ considerably in terms of protocol (e.g., fecal dilutions and respective multiplication factors), accuracy, precision, sensitivity, and need for financial, human and technical resources [10]. Although the McMaster (McM) method still remains one of the most widely used in veterinary medicine, new techniques have been developed in the last 20 years, like FLOTAC (FL) and Mini-FLOTAC (MF), with improved sensitivity (up to 100%) and precision (over 80%), in the diagnosis of GI parasitism in equids, ruminants, companion, exotic, and wild captive animals [7,19,20,21,22,23,24,25,26,27,28,29,30]. These FL and MFs’ improvements over the McM method may have the potential to increase horse owners’ willingness to shift towards surveillance-based control strategies instead of maintaining the unsustainable method of routine deworming [7].

The current study aimed to compare the analytical performance of the McM, FL, and MF techniques in the diagnosis of strongyle infections in horses kept at two Portuguese facilities, a horse stud farm and a veterinary faculty.

## 2. Materials and Methods

### 2.1. Study Design—Horse Populations and Fecal Sampling Procedures

**Figure 1:**
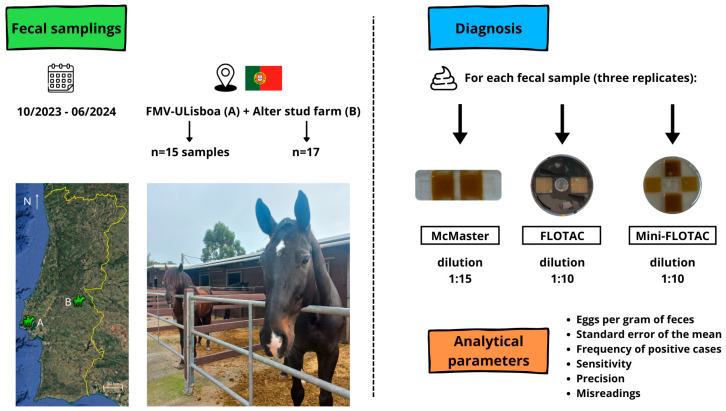
Illustration of the experimental design of this study, including fecal sampling and diagnostic procedures, and analytical parameters assessed (figure created using Canva, https://www.canva.com; Portugal map retrieved from Google Earth Pro, Google LLC; all other photos are original).

Between October 2023 and June 2024, a total of 32 fecal samples were collected at two Portuguese horse populations (Figure 1): 17 from the Alter Stud Farm (Alter do Chão; GPS coordinates: 39°13′19.8″ N 7°41′10.3″ W) and 15 from the Faculty of Veterinary Medicine of the University of Lisbon (FMV-ULisboa) (Lisbon; GPS coordinates: 38°42′50.77″ N 9°11′43.78″ W). There are no records regarding the deworming practices of the Alter Stud Farm and FMV-ULisboa in the last six years.

All samples from the Alter Stud belonged to Puro Sangue Lusitano (PSL) horses, aged between 4 and 13 years old (y.o.), whereas the samples from FMV-ULisboa included several breeds: PSL (*n* = 10; ages: 8–27 y.o.), Sorraia (*n* = 1; 10 y.o.), Dutch Warmblood (KWPN) (*n* = 1; 11 y.o.), Oldenburg (*n* = 1; 14 y.o.), Arabian (*n* = 1; 18 y.o.) and a KWPN-Gelderlander cross (*n* = 1; 22 y.o.).

Samples were collected immediately after excretion, and just from their superficial portion, and then transported in a cooling bag to the Laboratory of Parasitology and Parasitic Diseases of FMV-ULisboa, where they were stored at 4–5 °C for up to two weeks (maximum), before being processed.

### 2.2. Fecal Samples’ Processing—Modified McMaster, FLOTAC and Mini-FLOTAC Methods

All samples were processed using the McM, FL and MF methods, performing three technical replicates per sample, and the nematode eggs were identified based on the descriptions from Zajac and Conboy [31] and Thienpont et al. [32].

For the McM method, 2 g of previously homogenized feces were mixed with 28 mL of saturated sucrose solution (a specific gravity of 1.2) (a dilution of 1:15). The fecal suspension was filtered, transferred to an McM slide and visualized under a light microscope, using a total magnification of 100× [33,34]. The shedding values (eggs per gram of feces, EPG) were determined using a multiplication factor of 50.

The procedure for the FL method was adapted from the protocol established by Cringoli et al. [29] for ruminants and horses. Briefly, 5 g of previously homogenized feces was added to the Fill-FLOTAC device and mixed with 45 mL of tap water (dilution of 1:10); the fecal suspension was then transferred to test tubes and centrifuged at 1500 rpm for 3 min. After discarding the supernatant, the resulting pellet was homogenized with 6 mL of saturated sucrose solution (specific gravity of 1.2), and the suspension was added to the FL counting chambers, which were centrifuged at 1000 rpm for 5 min. At that time, the reading disk was rotated, and the chambers were visualized under a light microscope using a total magnification of 100×; the EPG values were determined using a multiplication factor of 1.

Finally, the protocol for the MF method followed the guidelines proposed by Cringoli et al. [30] for ruminants and horses: 5 g of previously homogenized feces was added to the Fill-FLOTAC device and mixed with 45 mL of saturated sucrose solution (a specific gravity of 1.2) (a dilution of 1:10). The fecal suspension was then transferred to the counting chambers and left to rest for 10 min on the lab bench. After the rotation of its reading disk, the chambers were visualized under a light microscope using total magnifications of 100× and 400×; the EPG values were determined using a multiplication factor of 5.

### 2.3. Statistical Analysis

The software Microsoft^®^ Excel^®^ (Microsoft Corporation, Redmond, WA, USA, 2024) was used for storing all parasitological data recorded in each horse population and for descriptive statistics. The mean EPG, coefficient of variation (CV), precision, and standard error were calculated for each set of replicates and each method, whereas the frequency of positive cases, sensitivity, and misreadings were calculated for each method. The means of the first three parameters determined their values per method, while the mean of the standard errors determined the standard error of the mean (SEM) per method.

The precision was computed by subtracting the CV from 100%, as described in Alowanou et al. [20], Noel et al. [35] and Bortoluzzi et al. [36]. The frequency of positive cases corresponded to the number of positive results divided by the total number of samples, while the sensitivity corresponded to the number of positives divided by the total number of true positives (i.e., samples that had a positive result for at least one of the techniques, considered as the gold standard [22]). The percentage of misreadings corresponded to the number of false negatives (i.e., negative reads for the technique, when at least one of the others tested positive) divided by the number of samples.

In samples where the EPG average values were equal to zero, their respective SEM and CV values were not included for statistical analyses; the same approach was used for negative precision results.

The software IBM^®^ SPSS^®^ Statistics (v27 for Windows; IBM Corporation, Armonk, NY, USA) was used to first subject global EPG, SEM and precision data recorded in this study to normality analysis, using the Shapiro–Wilk test (*n* < 50 samples). This allowed us to conclude that the EPG and SEM data were not normally distributed (*p* < 0.001 for both parameters, in all methods), whereas precision had a normal distribution (*p* = 0.06–0.12). These results determined the use of the Friedman test with a post hoc Wilcoxon Signed Rank test for EPG and SEM comparisons between techniques, the Spearman test for EPG correlations, the ANOVA test with a post hoc Tukey HSD test for precision comparisons, the Chi-Square test for the sensitivity and frequency of positive cases comparisons, and finally, Fisher’s Exact test for misreading comparisons. Cohen’s kappa statistics (k) was also determined to assess the agreement between the diagnostic techniques using the criteria according to Barda et al. [37]: poor (k ≤ 0), slight (k = 0.01–0.20), fair (k = 0.21–0.40), moderate (k = 0.41–0.60), substantial (k = 0.61–0.80), and almost perfect (k = 0.81–1.00). A significance level of *p* < 0.05 was used for all tests.

## 3. Results

Regardless of diagnostic technique, the three FEC methods enabled the detection of strongylid infections, the only group of GI parasites identified, in 84% (27/32) of the animals. The MF method detected the highest percentage of positive cases (78%), although it did not statistically differ from the other methods (*p* = 0.85) (Supplementary Table S1).

The McM method detected significantly higher strongyle egg shedding values (584 EPG, *p* < 0.001) in comparison to FL and MF (219 EPG, for both) (Table 1). Regarding the FEC dispersion, the SEM of MF and FL were approximately 5–7× lower and significantly different (respectively, 38 EPG, *p* < 0.001; and 25 EPG, *p* = 0.001) when compared to the McM method (179 EPG). FL also showed the highest analytical precision (72%), but it was only significantly different when compared to McM (52%, *p* = 0.03). Lastly, the MF method achieved the highest sensitivity (93%) and the lowest percentage of misreadings (3%), even though both results did not differ from the remaining methods (*p* = 0.90, and *p* = 0.69, respectively).

Finally, the three methods were found to be positively and significantly correlated with each other (Table 2), and according to Cohen’s kappa coefficient, shared substantial and statistically significant agreement with each other (Table 2).

## 4. Discussion

Horse strongyles have a direct life cycle: their eggs are excreted to the environment with the hosts’ feces and only then undergo larval development. This offers the opportunity of using feces as an indirect and non-invasive method for estimating the parasitism status within the host’s GI tract, with few exceptions, such as encysted L3/L4 larvae in the small intestine’s mucosa [4,38]. However, only a small percentage of the herd (the 80/20 rule) is responsible for the majority of the excreted eggs [5,25].

Reliable coprological diagnostic techniques, ideally with high sensitivity, specificity, accuracy, and precision, are essential to detect and monitor infections that may pose serious animal and public health concerns [9,20,29] while simultaneously identifying these high egg-shedders. This allows for a targeted and rational use of antiparasitic drugs, which, together with appropriate animal husbandry, make the foundations for an efficient and sustainable deworming program [7,9,21,39], capable of reducing reinfections and pasture contamination, improving the assessment of treatment effectiveness [25] and, hopefully, slowing down anthelmintic resistance.

The findings of this study are consistent with the existing literature, with FL and MF performing better than McM, but similarly to each other, reinforcing how both outperform classic copromicroscopic techniques, particularly in low infection intensity or multiparasitism settings [7,19,20,21,22,23,24,25,26,27,28,29,30]. The methods differed significantly in FEC, SEM, and precision: McM showed the highest FEC (584 EPG) of all methods, while both FL and MF showed lower SEM (25 and 38 EPG) and higher precision (72% and 65%) than McM.

These results can be explained by the protocols themselves. The protocol for McM starts with a smaller quantity of sample (2 g) and finishes with a more diluted fecal suspension (1:15) than the protocols of its counterparts (5 g and 1:10, respectively). Additionally, an McM slide analyzes a much smaller volume of suspension than an MF or an FL chamber (0.30 mL, 2 mL and 10 mL, respectively). These details are responsible for the 10–50-fold increase in the McM method’s multiplication factor (50), in contrast to MF (5) and FL (1) [7,9,21]. Unlike sample size or shedding levels, the greater the multiplication factor, the smaller the method’s precision [7,10].

Furthermore, the protocols for MF and FL involve the use of Fill-FLOTAC and the mechanical separation of floated eggs from debris by rotating the reading disk by 90° [29,30]. The former enables the integrated homogenization and filtration of fecal suspensions within a closed system, minimizing parasitic forms’ losses [30], while the absence of debris after the rotation of the disk improves visibility and thus the methods’ specificity and sensitivity [7,21].

Despite its improvements over McM, FL’s general use is constrained by the need for specialized equipment, such as a centrifuge adapted to its chambers, and its overall time consumption and labor intensity [7,20,24,25,28,30]. Since there were no significant differences between FL and MF across all parameters evaluated, MF appears as a viable alternative to FL for the diagnosis of equine GI parasitism, with the added advantage of not requiring centrifugation [7,19,20,21,22,23,24,25,26,27,30].

Although the results were not statistically significant, the MF method achieved a higher frequency of positive cases and diagnostic sensitivity, as well as a lower percentage of misreadings. Despite the non-significance, it is reasonable to assume there is a connection: a lower percentage of misreadings means there is a lower proportion of false negatives, which means there is a higher probability of an infected animal receiving a positive test result; in turn, this could translate into a higher frequency of positive samples than other methods. The lack of statistical significance is most likely due to the small sample size, so it would be interesting if future studies could take it into account and further investigate this with a bigger sample.

There are some external factors that could have also interfered with the overall results: the horses’ deworming history, the time between excretion and collection, and storage time before processing. The EPG values observed suggest that it is safe to exclude management effects on both horses’ populations (Supplementary Table S1), even though it must be recognized that this exclusion cannot be performed with full certainty due to the absence of deworming records. The samples were carefully collected immediately after excretion, so the time spent in the environment would not have been sufficient to significantly affect the tests’ results, both in terms of contamination and egg survival. Lastly, strongyle eggs can survive one to two weeks at 4–5 °C, and even though they can embryonate at 6 °C, the larvae cannot hatch [40]. The samples were stored in a refrigerated environment, never exceeding two weeks; therefore, it is safe to assume the storage time effects were not statistically significant on the methods’ overall performances.

## 5. Conclusions

The identification of parasitic infections in horse populations using a highly sensitive and precise technique is of the utmost importance for targeted selective drug treatments so that only horses with a higher FEC are treated. The overall results from this study reveal that the use of FL and MF methods detected strongyle infections with 5–7× less EPG dispersion in comparison to the McM method, with FL reaching the highest precision of analysis (72%). These results suggest the usefulness of implementing FL or MF methods (depending on the available resources) in equine medicine for precise and, in the latter case, quick parasitological diagnosis.

## Figures and Tables

**Table 1:** Performance results obtained with the implementation of the McMaster (McM), FLOTAC (FL) and Mini-FLOTAC (MF) techniques in the diagnosis of equines’ strongyle infections.

Technique(s)	FEC * (EPG)	SEM (EPG)	Frequency of Positive Cases (%, P/T)	Precision (%, 1-CV)	Sensitivity (%, P/TTP)	Misreadings (%, FN/T)
McM	584	179	72 (23/32)	52	85 (23/27)	9 (3/32)
FL	219	25	75 (24/32)	72	89 (24/27)	9 (3/32)
MF	219	38	78 (25/32)	65	93 (25/27)	3 (1/32)
McM × FL (*p*-value)	<0.001	0.001	0.85	0.03	0.90	0.69
McM × MF (*p*-value)	<0.001	<0.001		0.22		
FL × MF (*p*-value)	0.76	0.28		0.59		

* FEC—fecal egg count, EPG—eggs per gram of feces, SEM—standard error of the mean, P—positive cases, T—total number of samples, CV—coefficient of variation, TTP—total true positives, FN—false negatives.

**Table 2:** Spearman’s correlation and Cohen’s kappa agreement results obtained when comparing the McMaster (McM), FLOTAC (FL) and Mini-FLOTAC (MF) techniques.

Techniques	Spearman’s Correlation (r_s_)	*p*-Value	Cohen’s Kappa Agreement (k)	*p*-Value
McM × FL	0.96	<0.001	0.76	<0.001
McM × MF	0.92	<0.001	0.67	<0.001
FL × MF	0.93	<0.001	0.74	<0.001

## Data Availability

The data presented in this study are available on request from the corresponding authors.

## Supplementary Materials

The following supporting information can be downloaded at https://www.mdpi.com/article/10.3390/pathogens14111075/s1, Table S1: Equine Parasites Shedding and Frequency (Raw Data).

## Abbreviations

The following abbreviations are used in this manuscript:

CV	Coefficient of variation
EPG	Eggs per gram of feces
FEC	Fecal egg count
FL	FLOTAC
FMV-ULisboa	Faculty of Veterinary Medicine of the University of Lisbon
GI	Gastrointestinal
KWPN	Dutch Warmblood
McM	McMaster
MF	Mini-FLOTAC
PSL	Puro Sangue Lusitano
SEM	Standard error of the mean
y.o.	Years old
